# Medico-Chirurgical Transactions

**Published:** 1847-04-01

**Authors:** 


MEDICO-CHIRURGICAL TRANSACTIONS. Published by the Royal Medical and Chirurgical Society of London. Twenty-ninth Volume. Volume the Eleventh of the Second Series, 1846.

[Continued from No. IX., p. 233].

VII. AN ACCOUNT OF A CASE IN WHICH THE CORPUS CALLOSUM, FORNIX, AND SEPTUM LUCIDUM WERE IMPERFECTLY FORMED. By *James Paget*, F.R.C.S., Warden of the College, and Lecturer on Physiology at St. Bartholomew's Hospital.

The brain described in this paper was found in a girl 21 years old, who died in St. Bartholomew's Hospital with pericarditis, and who had presented, while under observation there, nothing remarkable in the condition of her mind. The appearances presented by the brain are carefully and minutely described by Mr. Paget. In the hope of obtaining some guidance in the obscure physiology of the corpus callosum, he made careful inquiries into all that concerned this patient's mind. He could "not find otherwise than that this girl's mind was one of the least remarkable kind. Her only peculiarity was vivacity, and a want of caution, shewing themselves in an habitual rapidity of action and want of forethought, deliberation and attention. Certainly she could not be regarded as unusually deficient in either moral goodness or intellectual power."

Instances in which the corpus callosum is deficient without any other serious wrong condition of the brain are rare. Mr. Paget states the chief facts of three other recorded cases: one related by Reil, a second by Mr. Solly, and the third by Mr. Chatto. These cases suggest some interesting observations on the anatomy, development, and functions of the corpus callosum. We have only space for the following remarks:—

"On the whole, these cases force us to think that the corpus callosum has its office as an organ for the highest operations of the mind. But of what part of the mind it is one of the ministers, and how its function is discharged, we have no evidence whatever. Its structure indicates that it is not a nervous centre, neither a source of nervous power, nor a first recipient of the influence of the mind: for it is composed wholly of nervous filaments, such as, elsewhere, have no higher office than that of conducting impressions, and are incapable of either originating, controlling, or diverting them. If, therefore, as a reasonable hypothesis, we adopt the general expression of the office of this and other commissures, that 'they serve to ensure unity or harmony of action between those parts of the brain between which they are placed,' we must use it as meaning, for the corpus callosum, not that it is a centre of action from which similar, and, therefore, harmonious influences proceed to each side; but that it is formed of conductors, by which a part on one side of the brain is informed (as it were) of the state of some part on the other side, and, probably, is induced into the same state. But, whatever may be meant by this and any similar forms of expression, they must be held as only hypothetical; there is no substantial evidence for their truth." P. 73.

Mr. Paget states, in conclusion, that many of the facts adduced in this paper afford support to the doctrine of the brain being a double organ, a subject to which attention has lately been directed by the vigorous writings of Dr. Wigan.

### VIII. Case of Aneurism presenting some Peculiarities; with Remarks. By *Prescott Hewett,* Esq., Lecturer on Anatomy at St. George's Hospital School.

G. B., æt. 31, was admitted into St. George's Hospital, in May 1839, with a pulsating tumour, of the size of a pullet's egg, in the left groin, about an inch below Poupart's ligament, and apparently connected with the common femoral artery. Sir B. Brodie applied, on the 30th of the month, a ligature to the external iliac artery, and all pulsation in the tumour ceased immediately. Peritonitis followed, but he recovered from it. The ligature separated on the 25th day; the tumour in the groin became solid and decreased in size, and the patient was discharged cured August 14th. At the latter end of November he was re-admitted on account of a return of slight pulsation, and a whirring sound in the tumour, which had slightly increased in size. By pressure with graduated compresses all pulsation and sound disappeared and the patient was once more discharged. In Nov. 1841 there was a slight recurrence of pulsation without increase in size and no plan of treatment was adopted. In January 1842, the tumour was observed to have increased in size, but no pulsation whatever could be detected in it. " From this period the tumour gradually but steadily increased in size for the ensuing twelve months, during which time it grew to the size of the egg of an ostrich; its surface was somewhat irregular, and softer in some parts than in others, although the tumour itself was perfectly solid. During the whole of the time neither pulsation nor sound of any kind could be detected in the tumour." In January, 1843, the tumour became stationary, and some time afterwards it began to diminish; the decrease in size continued until July of the same year, when the patient died of phthisis.

At the examination of the body, the tumour, when separated from the neighbouring parts, was of the size of a full-grown fœtus, slightly irregular on its surface, but perfectly solid throughout. Upon being cut into it presented the characteristic layers of coagulated blood observed in aneurisms which have been cured.

" The portion of the superficial femoral artery, about two inches, with which this aneurism was connected, was flattened, completely obliterated, and identified with the tumour, below which the cavity of the artery was filled with a coagulum, about an inch in length, somewhat smaller than the vessel, and adherent to it at its upper part only; the remaining part of this vessel presented a natural appearance. The whole of the common femoral was dilated to the size of the common iliac, and irregular on its surface, the irregularity depending upon some smaller dilatations superadded so the general dilatation, which extended up to the external iliac, at the point of the giving off of the epigastric and circumflex arteries. The internal surface of this dilated vessel was covered by thin layers of coagulated fibrine, which, beginning a little below the origin of the epigastric, were continued downwards into the upper parts of the superficial and deep femoral arteries. In the superficial femoral, these coagula completely blocked up the cavity of the artery; but in the deep and in the common femoral, they merely formed a lining to these vessels, a large and distinct channel being still left for the passage of the blood. This channel was, for the greater part, perfectly smooth, and lined by a membrane, of new formation, presenting all the characters of the serous coat of the artery, for which it might easily have been mistaken. Both the membrane and the coagula were, however, with a little care, detached from the internal coat of the artery, which did not appear to be diseased. These coagula did not pass further than half an inch down the deep femoral; below this, the coats of the artery presented a perfectly healthy aspect.

"The external iliac artery, from a little above the origin of the epigastric and circumflex to within a quarter of an inch of the common iliac, was completely obliterated, and reduced to the size of a small quill; the point at which the ligature had been applied was marked by a constriction, situated about an inch above the origin of the epigastric. The various branches given off from the external iliac, and from the common and deep femoral arteries, were much larger than natural." P. 78.

Mr. Hewett notices, as remarkable in this case, the long intervals which elapsed between the two periods, when the pulsation recurred; intervals during which the tumour presented all the appearances of being cured. He observes :—

"No abnormal distribution of the arteries having been found, the re-appearance of the pulsation is to be explained by the situation of the aneurismal tumour, which, when once the collateral branches were sufficiently dilated, became affected by the large current of blood brought into its immediate neighbourhood, —a current of blood sufficient to overcome, for a time, the efforts made by Nature to establish a cure, which she ultimately accomplished.

"The great increase in size which this aneurism presented, even after all pulsation and all sound had ceased in it, is a fact worthy of the attention of all practical surgeons. By this increase in size, and the other circumstances accompanying this aneurism, several experienced surgeons were led to the supposition that the tumour was of a malignant character, and supplied with large arteries, the growth of which had been checked for a time by the obliteration of the external iliac artery." P. 79.

Lastly, attention is called to two points of pathological interest, viz. the formation of the thin delicate membrane lining the common femoral from the blood, and the liability, should blood pass between the membrane and the internal coats of the dilated vessel, of the appearances being mistaken for what is termed a dissecting aneurism.

This is a case of interest, and well-related.

### XI. Case of Cyanosis of forty years' standing, depending upon Congenital Obstruction in the Pulmonary Artery, and Patulous Foramen Ovale. By *Robert J. Spitta,* M.B. Lond., House-Surgeon to St. George's Hospital.

The patient, a female æt. 40, of diminutive stature, pigeon-breasted and affected with cyanosis from birth, was suddenly seized with dyspnœa and partial syncope, accompanied at first with convulsions, and afterwards with intense pain referred to the epigastrium, loins and hypochondria. This condition continued 24 hours, when, after a slight struggle, she expired. On examination of the body, the principal change in the heart was hypertrophy of the right side, the right ventricle being as thick as the left ventricle, and the right auricle three times as thick as the left auricle. The foramen ovale was patulous.

"The pulmonary artery was so malformed at its root as to render an accurate description of it extremely difficult. Besides the three semilunar valves usually found there, it presented an adventitious membrane situated immediately above them, and stretched completely across the artery, like the diaphragm across the body. This membrane was a line in thickness, and perforated in its centre, not by a circular foramen, but by a mere slit two lines in length and a line in breadth, with margins of a red colour, and fringed with fibrine of the blood. The three semilunar valves were thrown up as they are naturally in a healthy pulmonary artery during the systole of the heart, and fixed in that position by the adhesion of (what would have been) their free borders to the adventitious membrane." P. 83.

Mr. Spitta infers, no doubt correctly, that the membrane which obstructed the pulmonary artery was a congenital malformation. He is of opinion that the circulation of imperfectly-oxygenated blood impaired no function so much as the formation of animal heat, of which, it appears, the patient was remarkably deficient.

### X. On the Ganglionic Character of the Arachnoid Membrane of the Brain and Spinal Marrow. By *George Rainey*, M.R.C.S.

Mr. Rainey observes, at the commencement of this paper—

"As I believe it has never been supposed by physiologists that there exists within the cavity of the cranium an especial provision of organic nerves for the supply of the cerebral vessels, corresponding to those which, in the abdomen, go from the cæliac ganglia to the vessels of the viscera,—the arachnoid serving as a membranous plexus in which they ramify,—the announcement of this view can scarcely fail to be received by anatomists and physiologists with extreme doubt. Under this impression I have considered it admissible, at the commencement of this paper, to state, that the views herein set forth are based entirely upon anatomical facts, which I have endeavoured to describe in such a manner as will most facilitate the verification of their accuracy: for this purpose, also, I have detailed these facts and discussed their physiological application nearly in the order they occurred, whilst occupied in their examination." P. 85.

It is plain, from the preceding statement, that it would be fruitless to attempt giving a condensed account of the facts and inferences drawn from them, which are detailed in this paper. It must be carefully examined with the assistance of the plates to be appreciated. The views of the author are novel and interesting, and cannot fail to attract attention. But it must be recollected that, in the microscopic examination of nerves, there are many sources of error; and of this no more striking example need be given than the recent controversy respecting the nerves of the uterus. It is, however, due to Mr. Rainey to say that he narrates his facts truthfully and faithfully. He states what he has really seen, and not what he wishes to see in order to support a preconceived theory, and he moreover fairly invites inquiry. It is a pleasure to meet with so honest and zealous an enquirer as Mr. Rainey, considering the little encouragement which is given in this country to the pursuit of microscopic anatomy and physiology.

XI. An Account of a Case of partial Double Monstrosity. (Ischiopage Symelien of Geoffroy Saint-Hilaire, Heteradelphia of Vrolik). By *William Acton*, Esq., Surgeon to the Islington Dispensary.

The subject of this curious case was a Portuguese child, six months old, exhibited in London during last Spring, and rendered sufficiently notorious by a placard not remarkable for its decency, in which the infant is characterized as " the Human Tripod, or three-legged child, and first Bipenis ever seen or heard of." The monstrosity is thus described by Mr. Acton.

" Below the umbilicus, and to the right and left of the mesial line, are two distinct penes, each as large as the penis of a child of six months old: their direction is normal. I may mention that water passed from both organs at the same moment, during the time that Dr. Cursham and Mr. Perry were examining the infant with me. Each penis is provided with a scrotum, the outer half of each scrotum containing one testicle, the inner half of the scrotum is far removed from the outer, and the two inner halves appear like another scrotum between the two penes. Between and behind the legs of the child, we see another limb, or rather two lower extremities united together in their whole length. The upper part of this compound limb is connected to the rami of the pubis by a short narrow stem half an inch in length, and as large as the little finger, apparently consisting of separate bones or cartilage, for, on moving the compound limb, at the same moment the finger is kept on the stem, crepitation is felt, but I could not detect any pulsation. Immediately beyond this stem, and concealing it, the compound limb assumes a size as large as the combined natural thighs of the child, and within the upper part irregular portions of bone may be felt (probably a portion of a pelvis and the heads of the thigh bones), which may be traced down, united together into one mass, to a leg of comparative small size, though still larger than either of the healthy legs, and terminated by a double foot in the position of *talipes*, with the sole turned forwards, and furnished with ten toes, the two great toes being in the centre of the others: the two outer toes on each side are webbed.

" When the child is placed on its belly, the spine and back present a perfectly normal appearance; the anus is in its usual situation; the functions of the bowels are duly performed. Viewed in this position, the compound limb assumes a roundness and fulness equal to the buttocks of a young child, and a slight depression is observed, as if for the anus. Tracing the limb downwards, we find only one patella, which is on the same aspect of the limb as the anus, the joint bends freely, and the compound extremity terminates as above described. This compound limb is quite motionless, the upper portion alone appears endowed with sensibility, its vitality seems low, as the toes have a blueish appearance; the upper portion, however, is of the same temperature as the body of the child." P. 105.

Mr. Acton has not been able to meet with any recorded case analogous to this. He discusses the question of removal of this compound limb, and is of opinion that every circumstance is in favour of an operation. He particularly notices the low vitality of the limb, observing that, with every precaution that can be taken, the toes have now a blueish appearance, and the history of partial double monstrosities shows, that any, however slight, scratch or contusion heals slowly, and generally ends, at first, in the death of the part, and subsequently of the child. " If the infant escapes this source of danger, its system is found incapable of supporting this additional limb, and the child perishes from debility. There can then, I think, be no doubt that an operation will be necessary, to give the child a chance of arriving at puberty; and, in the absence of any one counter-indication, I think all will agree, the sooner this is performed the better, for the security of the child."

This child has since been exhibited in Paris, and we have noticed in the journals that more than one eminent French surgeon has expressed an opinion favourable to an operation. This paper is illustrated by an indifferent lithograph of the monstrosity.

XII. Some Remarks on Wounded Arteries, Secondary Hæmorrhage and False Aneurisms. By *Robert Liston*, F.R.S., Surgeon to University College Hospital.

The object of the author in this paper is clearly to justify the practice pursued by him in the treatment of the case of the late Mr. Seton, who died after a wound received in a duel at Portsmouth. This gentleman was 28 years of age, exceedingly corpulent, and had lived excessively freely for a series of years. He was wounded by a pistol bullet on the 20th of May 1845. The ball entered the upper and outer part of the right thigh and passed out in the middle of the fold of the left groin, thus traversing the course of the femoral vessels. The flow of blood was described to have been most impetuous and profuse, the blood being thrown in jets to a considerable distance—it was said, two or three feet. The patient was found by Mr. Jenkins of Gosport in an almost lifeless state, and he was with great difficulty recovered from the syncope and depression. A considerable swelling soon supervened over the lower part of the abdominal parietes from extravasation of blood.

May 27th. The seventh day from the receipt of the injury the swelling in the right groin began to increase in size, and distinct pulsation was then perceived in it. The tumour went on increasing gradually, and it had gained much more in bulk on the tenth day, when Mr. Liston first saw him, than it had done on any of the preceding ones. It was of an oval form, and elastic but firm, as if it was partly made up of coagulum and liquid blood. Pulsation was strong and distinct in all its parts. The opening on the right hip was filled up with a dry depressed slough; the wound in the left groin, a jagged slit, was closed by a very thin cicatrix. The patient's countenance was blanched and waxy, and his pulse quick and feeble. He had, in short, all the appearance of a person who had lost a great quantity of blood.

The nature of the case, Mr. Liston states, was very apparent. A large false aneurism, not well bounded, rapidly increasing and arising from a wound of the femoral artery, or of some branch divided close to its origin, had to be arrested, otherwise the patient must be left exposed to the risk of perishing suddenly and at no distant period. After consultations on the evening of the 30th and morning of the 31st, the external iliac artery was tied with the loss of not more than a table-spoonful of blood, and with the immediate effect of arresting the pulsation, and removing, in a great measure, the tension of the tumour. Symptoms of peritonitis supervened the same evening, and on the following afternoon the patient sunk.

Mr. Liston subjoins the following account of the post-mortem examination by Dr. Allan of Haslar. The course of the bullet was traced from the outside through a dense layer of fat, about two inches in thickness. It had divided one of the superficial branches of the femoral artery, about half an inch below Poupart's ligament, and about an inch from the main body of the femoral artery, which had caused a false aneurism. The sac contained about three ounces of blood. No other artery appeared to have been wounded. A considerable quantity of sero-purulent fluid was found in the abdominal cavity, and patches of acute inflammation were observed on the intestines. The peritoneum adjoining the wound of the operation was inflamed. It had not been injured by the knife. The ligature had been properly applied to the external iliac artery. The abdominal viscera were healthy, but loaded to an extraordinary degree with fat. There was some enlargement of the right limb, but apparently no mortification. The femoral artery was pervious. "The course of the ball was through a bed of fat, fourteen inches in length and three inches in depth, over the pubes, and no muscular substance was injured." The blood in the aneurismal sac was firmly coagulated, and there was no mark of recent oozing from the injured artery. The ball had passed immediately over and along the course of the artery, for about half an inch before dividing it. "It was given off from the fore part of the femoral artery, about half an inch below Poupart's ligament, and passed in the direction of the pubes. Between its origin and division there was not more than an inch of extent. I raised it with the forceps from its attachment in the bottom of the wound, from the part where it was divided, to where it passed through the fascia, down to the femoral artery; and, although it was not actually detached, it would not have borne a ligature."

Mr. Liston remarks, "that a vessel of this class should have bled so furiously in the first instance, could not have been anticipated. Having done so, one can so far understand the active pulsation and rapid extension of the tumour." We question very much the accuracy of the account of the furious hæmorrhage in the first instance. It was not witnessed by any surgeon, and we well know the proneness of byestanders to exaggeration in respect to bleeding wounds, and the description of the hæmorrhage given in the paper, it is evident, cannot be relied on. It was a deep wound in a fat subject, and the external opening did not correspond with the divided artery, for it is stated that "the ball had passed immediately over and along the course of the artery, for about half an inch before it divided it." The rent in the clothes made by a bullet is small, and the clothes being loose, this rent was not likely to correspond so exactly with the wound as to admit the passage of blood in an uninterrupted current, and yet we are told, that the blood was "thrown in jets to a considerable distance—it was said, two or three feet," an occurrence which, under the circumstances, we believe to have been improbable, if not impossible. Mr. Liston adds—

"It was, of course, quite impossible in this case to determine whence the blood flowed into the aneurismal cavity. The principal vessel going to the limb might, for what we knew, have been wounded, or some considerable branch, as the epigastric, the external circumflex, the external pudic, or perhaps a considerable common trunk sending off the superficial epigastric, and branches to the inguinal glands, near their origins from the iliac, or common femoral. The division of even a small branch close to the principal vessel, it is well known, pours out blood furiously, as much so, in fact, as if an opening in the coats of the artery itself were, so to say, punched out, corresponding in size to the area of the branch. 'The division of an artery of the size of the last referred to (small muscular branches, which spring from most arteries at irregular intervals), at a distance from the source from which it springs, is of little importance. It contracts, and soon ceases to bleed. But when it is divided close to the trunk, blood issues from it, as it would if an opening, equal in size to the calibre of the little branch, were made in the trunk itself.'—Quain's anatomy, 5th Edit. p. 595. This I have actually experienced in operating on the femoral artery in the groin, as will appear by and by." P. 112.

The observation here quoted from Mr. Quain clearly applies to a vessel divided close to the trunk, in fact immediately as it comes off from it, and is true enough, as no contraction of the orifice can occur, since the vessel divided is entirely removed, and the opening is equivalent to one made in, or punched out, of the coats of the main artery. But the observation is totally inapplicable to Mr. Liston's case. Dr. Allan, who made the post-mortem examination, states that the superficial branch was divided about an inch from the main body of the femoral artery—which is a very different affair from a wound close to the trunk. The coats of a small superficial artery, divided an inch from the trunk, may not only contract so as to arrest all bleeding, but have sufficient space for the formation of a firm coagulum. Indeed, we contend that, if the wound had occurred at the distance of even a quarter of an inch, the coats of the vessel would still have been capable of the contraction which ordinarily occurs in these small vessels. It seems to us, that Mr. Liston completely fails in offering a satisfactory explanation of the furious bleeding and the formation of the large false aneurism. We think that this eminent surgeon made an erroneous diagnosis, in concluding that a large vessel, instead of a small branch, was wounded—and we express this opinion with no view of ascribing blame to him on that account, considering that he had only imperfect and second-hand information—the difficulty of making a satisfactory examination, in so fat a subject, the apparent course of the ball, and the depressed condition of the patient, the conclusion may be justified by the circumstances of the case, and be such as would have been arrived at by any other skilful surgeon. But we do blame Mr. Liston for not fairly admitting his error—for endeavouring, by bad reasoning and special pleading, to convince us that it was no error at all, and that the effects of a division of an insignificant branch was as dangerous as a wound of the femoral artery itself.

Mr. Liston, having made up his mind as to the nature of the case, pursued what, if he had been correct in his conclusion, would have been the right practice, and a course which, under the circumstances of the present case, we think no surgeon who has experienced the difficulties of practice would be hasty in condemning. It has been stated that the operator ought to have opened the sac and secured the wounded vessel by ligature, and we have no doubt, notwithstanding the tenor of his remarks, that, had he been able to ascertain the exact nature of the case, he would have done so, but, under the impression that the femoral itself or a large branch was wounded, he rightly urges that this would not have been safe practice, remarking that patients who have lost a great quantity of blood are often lost by the effusion of even a small quantity, and, after adducing some cases in point, he observes, that the danger must "be very much enhanced, when the tumour is so placed that there is no possibility whatever of making pressure on the trunk of the artery on the proximal side of the opening into it, or of the origin of any branch wounded close to where it is given off. In the case of Mr. S., the principle of tying the artery, where wounded, could not possibly be carried out with any propriety. It was not known what vessel was really implicated, and this could not be known during life. If the tumour had been lower down, somewhere in the thigh, the question of cutting into the sac might have been entertained." We are fully ready to admit that Mr. Liston adopted a proceeding—a bold one it is true, which may be justified by the *apparent* circumstances of the case. But he goes on to remark :—" As it was, the cyst could not have been opened without great loss of blood, however dexterously and boldly it might have been set about. Even knowing, as we do now, from whence the blood came, it was not likely that the application of a ligature to the branch would have permanently arrested the bleeding. Indeed, it is distinctly stated by Dr. Allan that the vessel would not have held a ligature." Now, after knowing the result of the post-mortem examination, that merely a superficial branch of the femoral artery was wounded at the distance of an inch from the main trunk, and that there were only three ounces of extravasated blood found in its vicinity, we can see no good reason why the sac might not have been laid open and the wounded vessel tied without any difficulty whatever. Dr. Allan does not state why the vessel would not have held a ligature, and we cannot conclude that it was too much injured to have borne one, because, in that case, it would be very unlikely to have bled, as every pathologist knows that a vessel torn across, and much lacerated or contused, rarely indeed pours out blood.

Mr. Liston observes that, the placing of a ligature upon the external iliac, so as to favour the coagulation of the contents of the tumour and the permanent closure of the wounded vessel, seemed to afford the only chance of safety, and he asks if there is anything to bear a surgeon out in adopting this practice ? " Can any cases be brought forward to show that vessels bleeding outwardly, or pouring their contents into the tissues of a limb or region, have ever been put in a way of being permanently closed, in consequence of the flow of blood being intercepted and weakened for a time, by the application of a ligature upon the principal arterial trunk ?" Every surgeon who has had any experience must have witnessed cases of this kind. Mr. Liston adduces three cases of wounds about the hand, attended with secondary hæmorrhage, in which the practice succeeded, though he admits, at the same time, that the practice cannot be expected to be uniformly successful, and mentions two cases in which bleeding returned after ligature of the brachial artery. The proper treatment to be adopted in such cases is no doubt an important practical question, but we cannot see that it applies in any way to the case of Mr. S., because, in this instance, had a large vessel been wounded, as Mr. Liston expected, instead of an insignificant one, compression of the main trunk above the wound was impracticable. For the same reason we shall not detain our readers with the treatment of secondary hæmorrhage from stumps, by ligature of the principal artery of the limb, cases of which are mentioned by the author to support the practice he pursued. He states—

"A few cases may be added in which secondary hæmorrhage followed wounds and ulcerations of arterial trunks and branches, and others in which false aneurisms formed, and where the practice of securing the trunk at some distance by impeding for a time the flow of blood to the part, gave opportunity for the curative process of nature to close the wounded vessel permanently, so that the lives of the patients were thus preserved." P. 122.

Some of these cases are to the purpose, and are of considerable interest, but our space only permits us to quote one of them, the last.

"It is to be found in the works of a well-known army surgeon. A man, 30 years of age, was wounded on the glorious 18th of June, by a musket-ball, which entered the left groin a little below Poupart's ligament, and passed through the inside of the thigh.

"'On the 29th, (mark, the 10th day from the injury), the slough from the anterioi wound came away, and was followed by so frightful a hæmorrhage as to leave no doubt whence it proceeded, nor (from the wound being so high up) any alternative as to the means to be adopted for stopping it.'

"The external iliac was tied, but the patient died of fever on July 5th: there is, however, no account given of any dissection of the parts.

"'In this case the necessity of the operation is evident, and as far as it went, also, its success. Not a drop of blood was lost after it.'—So says Mr. Guthrie, who, it is to be presumed, conducted the treatment of the patient. We are here left to conjecture what vessel was really wounded. It is said 'that there could be no doubt as to whence the hæmorrhage proceeded;' but it may have been from a branch, as likely as from the trunk, and those who have attended to the description of the hæmorrhage in the case of Mr. S., to its impetuosity, and amount, will bear me out in this assertion. The external pudic, or external epigastric, or one of the circumflex arteries, may have been here cut off close to their origin, just as likely as that the femoral was wounded." P. 128.

The significant manner in which this case is mentioned seems to imply, what indeed his writings fully show, that Mr. Guthrie condemns the practice recommended and adopted by Mr. Liston, viz. securing the main artery at a distance from the wound on account of secondary hæmorrhage, instead of the injured vessel itself. We cannot now discuss this question, and, as before stated, it has no direct bearing on the case of Mr. S. Mr. Liston concludes:

"I have thus endeavoured to show—

"1st. That the case of Mr. S. was one of great and immediate danger.

"2ndly. That some decisive step required to be taken, and that without a day's delay, in order to avert the danger that impended over him.

"3rdly. That very great risk would have been incurred in searching for and attempting to put a ligature upon the wounded part of the vessel.

"4thly. That there is ample authority for adopting the step which was had recourse to in the case. Ligature of the external iliac was undertaken, in order to ward off the expected fatal termination, through a sudden escape of arterial blood." 129.

We, on the contrary, have endeavoured to show that Mr. Liston mistook the case—that it was not one of such great and immediate danger as he supposed, or as the history and symptoms led him to infer—that no operation or decisive step was really required—that no particular risk would have been incurred in placing a ligature upon the wounded vessel —and, we may add, that there is no authority whatever for applying a ligature to the external iliac for a wound of a superficial branch of the femoral. To say that there was any actual danger of a fatal termination, through a sudden escape of arterial blood from a vessel of the size of that wounded, is too absurd to require serious refutation—the *expected* fatal termination was part of the erroneous diagnosis which led to the performance of a severe, dangerous, and unnecessary operation.

Mr. Liston, by the publication of this case and the elaborate manner in which he has endeavoured to justify his treatment of it, has fairly challenged the comments which have been made upon this paper. In the imperfect and uncertain state of our art, ample allowance must be made for errors, too often unavoidable, in diagnosis and treatment. But what we feel bound to censure is the attempt to gloss over the mistakes of practice, and, after the post-mortem examination has exposed the error and revealed the true nature of the case, to maintain the necessity for a proceeding erroneously adopted. If the example were followed, much evil might result, and it is a poor compliment to the intelligent race of medical men of the present day to presume that they cannot penetrate the veil of bad pathology and special pleading by which the necessity for the operation is defended. We sincerely regret that so eminent an operating surgeon should by such means mar the reputation he evinces so anxious a desire to support.

### XIII. A Case in which a large Tumour was developed in the Substance of the Fifth Nerve and its Ganglion. By *James Dixon*, Esq.

This paper may be considered as a supplement to one published in the preceding number of the Transactions (vide our Number for April, 1846, p. 438), consisting of an account of the morbid changes in the brain of the patient, a woman aged 59, who had paralysis of the fifth nerve on the left side and had lost the left eye. The parts supplied by the facial nerve on the same side became paralysed, there was total deafness of the left ear and, after attacks of giddiness and loss of memory, she died. The temporal muscle on the right side was of a natural appearance, whilst the left one was so wasted as hardly to be recognised. Everything was healthy in the brain and nerves on the right side. To the left of the pons varolii was an oval mass, slightly attached to the encephalon at the junction of the pons and crus cerebelli, and extending forwards, beneath the dura mater as far as the foramen lacerum orbitale. This tumour, from which the three divisions of the fifth nerve emerged, had hollowed out for itself an irregular pit in the concavity of the great wing of the sphenoid bone. The morbid growth proved to be a degeneration of the trunk of the 5th nerve and Casserian ganglion. The chief alteration in the eye was adhesion of the iris to the lens, which was of a pale yellow and opaque at the centre. Mr. Dixon remarks :—

"It would be absurd, from a single case like the present, to generalize upon the influence of different nerves in nutrition; but it will be observed that there was here no atrophy of the eye-ball, although the Casserian ganglion, and the whole trunk of the fifth nerve between that body and the brain, had been replaced by an adventitious growth." P. 134.

XIV. On the Capacity of the Lungs, and on the Respiratory Functions, with a view of establishing a precise and easy Method of detecting Disease by the Spirometer. By *John Hutchinson,* Surgeon.

Mr. Hutchinson commences by reviewing the speculations and opinions of those who have preceded him in treating of the mechanism and functions of the lungs. He begins with Hippocrates, who reckoned air one of the elements of life; attributes to Galen a recognition of the fact that the thorax distending draws in the air, and that the lungs follow the dilatations of the chest, and sums up the opinions which, during 1500 years, from Galen to Robert Boyle, were received by different naturalists and philosophers, under the three following heads :—

"First,—'That by the dilatations of the chest, the contiguous air is thrust away, and that, pressing upon the next air to it, and so onwards, the propulsion is continued till the air is '*driven* into the lungs' and so dilates them.'

"Second,—That the chest is like to a pair of common bellows, 'which becomes to be filled because it is dilated.'

"Third,—'That they are like a bladder, which is therefore dilated because it is filled.'" P. 139.

Torricelli, the pupil of Galileo, discovered in 1643 the law of atmospheric pressure, and explained why air enters the lungs in inspiration. Fabricius described the action and properties of the diaphragm, Malpighi and John Templer the structure of the lungs. Borelli, Juvin, and Dr. James Keill in succession made admeasurements of the quantity of air expired from the lungs; and in 1757, Black, Rutherford, Lavoisier, Priestley and Scheele, by discovering the composition of the atmosphere and of the respired air, threw much light upon the chemistry of respiration.

The author, having adverted to the propriety of considering respiration under two grand heads, as chemical, and mechanical; directs our attention to the latter of these.

He recognises in the varying capacities of the chest,—"first, extreme expansion or enlargement; second, extreme contraction or diminution; and third, an intermediate condition, an ordinary state."

We proceed to an explanation of the terms Residual, Reserve, Breathing, Complemental Air and Vital Capacity, to each of which he has affixed a specific meaning, likely henceforth to be always associated with it.

Residual air then is that air which remains in the lungs after the most complete expiration that it is in the power of the individual to make.

Reserve air is the difference between the quantity remaining after easy or ordinary expiration, and the residual air.

Breathing air is that quantity which enters and leaves the chest in ordinary respiration.

Complemental air is the difference between the quantity of air contained in the chest after the most forced, and that contained after ordinary inspiration.

The vital capacity is made up of the reserve, breathing, and complemental airs together, or, which is the same thing, it is the difference between the aerial contents of the chest during extreme expansion, and during the most forcible contraction. In all cases the air is measured at 60° Fahr.

The residual air is independent of the will, and always present in the chest. The reserve air, to use a simile, is a tenant at will. The breathing air is constantly passing in and out. The complemental air is seldom in the chest and never is so long.

We pass over the details of particular measurements of these several quantities of air and give only the general results with Mr. Hutchinson's pertinent remark upon them.

| | | | | | |
|---|---|---|---|---|---|
| " Residual air ranges from | - | 40 | to | 260 | cubic inches. |
| Reserve air | - - - | 77 | to | 170 | do. |
| Breathing air | - - - | 3 | to | 100 | do. |
| Complemental air | - - | 119 | to | 200 | do. |
| Vital capacity | - - - | 100 | to | 300 | do. |

" This forms the basis of our present knowledge, from which I can only gather that observers differ. It is possible that all these experiments may be correct; but allowing this, we cannot thence definitively solve the problem respecting the different quantities of air passing through the lungs." P. 150.

It is subsequently stated that these apparent discrepances arise from the absence of all correction for temperature and for difference of sex, height, weight, or age of the individuals examined.

The object of Mr. Hutchinson's researches being, by means of his instrument called the Spirometer, to ascertain the vital capacity of different individuals, in order to derive indications with respect to their health; it is necessary in the first instance to determine all the circumstances affecting its quantity, or the laws to which it is subject. Now this vital capacity is disturbed directly, or modified by the height, weight, and age of the person examined, as well as by disease.

In reference to the influence of the first of these, Mr. Hutchinson, from experiments made on nearly 2000 individuals, has deduced the law, that the vital capacity increases by 8 cubic inches for every additional inch of height for all statures between five and six feet; the average vital capacity for men of five feet being 174 cubic inches; or the bulk of air which at 60° has that measurement.

The effect of a considerable excess of weight beyond what may be considered to correspond to the stature of the individual is to diminish the vital capacity which is normal for that stature. Now the weights of 2600 men having been taken, it was found that 120 lbs. being the average of those who were 5 feet 1 inch high, 174 lbs. was that of men of 5 feet 11 inches. A near approximation then to the average normal weight for any intermediate height may be obtained by adding 5·4 lbs. to the former of these weights for each additional inch of height. It does not appear that a reduction of weight below this normal standard produces any alteration in the vital capacity ; and it seems that we may have an addition of weight to the extent of about seven per cent. beyond these averages without affecting it. But beyond this, the vital capacity will diminish at the rate of one cubic inch per lb. up to 35 lbs. weight of increase as a consequence of the impediments to respiration occasioned by corpulency. And for still greater weights a greater decrement of vital capacity per lb. of weight must probably be allowed.

As regards the effect of age, we find that vital capacity is increased from 15 to 35 years of age, and from 35 to 65 years of age it is decreased in the progression of 19, 11, and 13 cubic inches for the several successive decads.

"To conclude this portion of the inquiry, it may be added, that the healthy vital capacity is chiefly affected by three circumstances—height, weight, and age.

"By height, an increase of 8 cubic inches at 60° for every inch of height.

"By weight (at the height of 5 feet 6 inches), the vital capacity is not affected under 161 lbs., or 11½ stone; but above this point it diminishes the vital capacity in the relation of 1 cubic inch per lb. up to 196 lbs., or 14 stone. And at other heights, between 5 feet 1 inch and 5 feet 11 inches, ten per cent. may be added to the mean height, before we allow the weight to affect the vital capacity in the relation of 1 cubic inch per lb.

"By age (from 35 to 65), a decrease of rather more than 1 cubic inch per year." P. 174.

It is a curious and interesting result of numerous experiments, made by means of plaster casts of the thoracic cavity after death and subsequent to the removal of the heart and lungs, that the absolute capacity of the chest bears no fixed relation to the vital capacity. The conclusion that universal adhesions of the opposed surfaces of the pleuræ, do not diminish the vital capacity, is, if it be safe to trust to a single observation, an important step in knowledge, contrary as it is to all preconceived ideas on the subject.

A study of the phenomena attendant on respiration leads our author to the belief that the *ordinary breathing* movement is abdominal, caused by the descent of the diaphragm pushing out the abdominal viscera ; that the *deep inspiratory* movement is not so, but quite the contrary ; in this case, the sternum advances while the abdomen recedes; the chief enlargement of the thoracic cavity in deep inspiration being made by the ribs and not by the diaphragm.

In females, however, the ordinary breathing is thoracic, very little abdominal movement being perceptible.

The following paragraphs embody the chief inferences drawn from observations too numerous for us even to sketch in detail.

"Having now given a brief outline of the respiratory movements, I must recall the attention to the vital capacity.

"We have seen that this corresponds with the height, and not with the absolute capacity of the thorax: why is this the case? I confess myself as much at a loss to explain it as I was the first day I commenced the research. I believe the vital capacity is mathematically commensurate with the range of mobility or thoracic movement; but why the mobility increases in arithmetical progression with height, which it appears is chiefly dependent on the length of the limbs, and not on the length of the trunk of the body, I am incapable of explaining. So completely is mobility, and consequently the vital capacity, affected by nature, that a man will breathe in different positions different quantities of air: thus, standing, I blow 260 cubic inches; sitting, 255; and when recumbent, (supine) 230, (prone) 220; position making a difference of 40 cubic inches." P. 197.

The question of the relative force of the inspiration and expiration has received the attention of Mr. Hutchinson, and by means of an instrument fitted to the nose, and so contrived that he could measure by a column of mercury the suction power of the former, and the forcing power of the latter, he has shown that the opinion previously entertained is entirely erroneous. He remarks upon his results set forth in a tabular form: "It will be observed that the figures on each side of the same word differ in their value, the expiratory side ranging about one-third higher, because the power *manifested* (I do *not* mean power exerted) by these muscular efforts varies in this relation. Thus, a man capable of elevating by his inspiratory muscles 3·5 inches of mercury, may be expected to raise by his expiratory muscles 4·5 inches." He says, farther on, "I am inclined to look for the first intimation of debility from disease in the inspiratory effort, and not in the expiratory; the expiratory muscular effort may however be taken as a test of health when it exceeds the inspiratory."

The excess of the force of expiration over that of inspiration is attributed to the elasticity of the ribs and lungs co-operating with the former but opposing the latter power. Experiments were made to determine the amount of this force of elasticity, but our space prohibits our entering into a detail of them: they seem to show that it is very considerable.

There are some observations regarding the effects of decussating, diametric and oblique power which are in the main erroneous, as is the inference deduced from them to the effect that, every external intercostal muscular lamella can raise a rib independently of the lamella next above it. This matter, however, has little bearing on the general object of the paper, and like some others subsequently touched upon may be deemed episodical, and might perhaps have been reserved with advantage for a separate essay.

Mr. Hutchinson has, as we have seen, given guides for the determination of the healthy vital capacity in any individual as influenced by height, weight, and age; we are thus furnished with a standard of comparison, so that when, by means of the Spirometer, the actual vital capacity is ascertained, we can at once recognize the existence and the amount of any deficiency. In a table, in which are given the results of an examination of many phthisical patients, it is shown that, even in an early stage, the indications of the instrument are very decided, and important. It must be remarked, however, that the deficiency of vital capacity indicated by the instrument, depending as Mr. Hutchinson has shown upon diminished mobility of the thoracic walls, will not point out the specific disease. So far from this that his method has been found available by the author for the detection of ailments which might be supposed to be little capable of affecting the respiration, as for instance hernia, and ruptured tympanum. It was, however, the mercurial instrument before spoken of for measuring the force of inspiration that led to the discovery of these affections.

The Spirometer consists essentially of a cylinder closed at one extremity, inverted in another similarly formed, but of somewhat larger diameter and filled with water. Air is blown, by means of a pipe or tube, through the bottom of the larger, so as to come within the mouth of the lesser, and as this is exactly counterpoised by weights suspended from above, its elevation will indicate the quantity of air introduced reduced to the temperature of the water by its passage through it, and sustaining only the pressure of the surrounding atmosphere. In principle the Spirometer exactly resembles a gasometer, the form however is somewhat modified, and the dimensions are vastly reduced in the former.

In using the instrument, the person whose vital capacity is to be ascertained should be in a standing posture, and after inspiring as deeply as possible, should make as long-continued an expiration as he can through the tube, which is furnished with a proper mouth-piece. This process is to be gone through three times consecutively, the instrument being intermediately adjusted, and the greatest of the three results should be recorded as the correct one. The repetition of the trial is necessary in order to exclude the errors which may easily affect the conclusion from a single one. The posture is important, at least it appears from what has gone before, that if a person can only use the instrument while sitting or lying, allowance must be made for that circumstance. The deficiency of vital capacity being ascertained ; the inference that disease exists somewhere seems established with a painful certainty. The lungs we conceive will be found in fault in the vast majority of instances; and as there is no one of their diseases which is apt to have so long a period of latency, and afterwards to be so obscure, and doubtful in its progress as phthisis often is, it is especially upon this disease that the Spirometer is calculated to throw light. Subsequent experience, in two or three cases, mentioned in the paper, verified the melancholy anticipations of the author, where consumption had not previously been suspected. Farther experience of the reliance to be placed upon this method of examination will, in all probability, constitute the Spirometer a valuable instrument in the hands of medical men. Our best ground of hope of combatting phthisis successfully is found in our being enabled to recognize it at its commencement with certainty, so that we may attack it early and act against it with earnestness. Now the Spirometer promises to give us this advantage, and we shall watch its future use in diagnosis with much interest, and hopefulness.

No one can read Mr. Hutchinson's Essay without at once perceiving that he has not spared time, study, labour, or expence in his endeavour to give completeness to his researches upon the subject of respiration. He has multiplied experiments to so large an extent as to defy distrust in the general accuracy of his conclusions. He has incidentally, and collaterally to his main object, established many curious and interesting facts in connexion with the powers and mechanism by which the process of breathing is carried on ; the influences and derangements to which it is liable. He has made one direct application of the laws which he has discovered to the uses of practical medicine, at the same time that he has improved our knowledge of the mechanical conditions of respiration as a whole. Whether we consider the importance of the function which he has investigated, the probable advantages arising from his discovery, or the manner in which his researches have been carried on, we think him well entitled to the best thanks of his professioual brethren.

XV. Hydatid Cyst, either originating in, or pressing upon, the Prostate Gland. By *George Lowdell,* late House Surgeon to the Sussex County Hospital.

In this case, retention of urine and death were produced by a large hydatid cyst, which pressed upon the prostate and displaced the urethra. There is an appendix to the paper containing the particulars of another fatal case of retention of urine, occasioned by a cyst containing hydatids developed in the pelvis, communicated to the Society by Mr. Curling. In both instances there were hydatids in other parts of the body. The rarity of hydatid cysts in the pelvis, and their serious effects, render these cases well deserving of record.

XVI. Cases of Varicocele treated by Pressure, with Observations. By *T. B. Curling,* Lecturer on Surgery, and Assistant-Surgeon at the London Hospital.

Mr. Curling states that, "three years ago, a case of varicocele, cured by the application of pressure to the spermatic veins, came under my notice, and being struck with the peculiar adaptation of this plan of treatment, to counteract the injurious effects of the dilated veins, I determined to give it a trial. In a work on the Diseases of the Testis, which appeared a few weeks afterwards," he stated the object of this method of treatment to be "the maintenance, whilst the patient is in the upright position, of such a degree of pressure on the spermatic veins as may be sufficient to relieve them from the superincumbent weight of the blood, without at the same time endangering the integrity of the testis, by obstructing the spermatic artery, and without causing so much uneasiness as to render the remedy as painful as, or more difficult to be borne than, the disease. This pressure must be continued a sufficient time, to enable the coats of the vessels to return to their natural dimensions, and to acquire strength to carry on the circulation. When this is effected, the patient is cured." He also remarked, "I look forward with no slight interest to the result of further trials of a remedy, which seems to me to be based on sound views of the pathology of the disease," and that this "plan appears to be particularly applicable to cases of varicocele in young persons, whose reparative powers would be sufficient to restore the veins, when relieved of pressure, to a healthy state." Since these observations were written, Mr. Curling informs us that he has treated many cases of varicocele by pressure; and, as a sufficient period has elapsed to enable him to form a just opinion of the value of this plan of treatment, and of its advantages over other methods, he here submits the results of his experience in the management of this complaint to the consideration of the Fellows of the Society. Mr. Curling gives the particulars of three cases, in which firm, steady, and continued pressure on the spermatic veins at the external abdominal ring succeeded in curing the disease. They seem satisfactory examples of permanent benefit derived from the treatment pursued. Mr. Curling observes that, to these cases of cure by pressure, he could add two other cases, if necessary, to establish the value and utility of this plan of treatment, besides the case mentioned in his work on the Diseases of the Testis, and another case of a gentleman, aged 27, who was affected with a rapidly increasing varicocele, for which he had worn a truss two months with benefit, when he quitted this country for Canada. He returned to England at the expiration of three years with the varicocele quite cured. He had left off the truss after wearing it fifteen months.

Mr. Curling states—

"In the above cases, the dilatation of the veins had taken place at a comparatively early period of life, was not excessive, nor in two of them of long duration, but was productive of more or less inconvenience and uneasiness, which could be only partially, or scarcely at all, remedied by the suspender; they were precisely the cases in which it was presumed that pressure, by relieving the veins of the superincumbent weight of the blood, would enable their coats to recover their proper size and tone.

"The same method of treatment has been applied to several other cases of varicocele, of a like character to the above, in some of which, after the patients had derived so much benefit from it, that hope was entertained of a permanent cure being effected, they ceased to remain under my observation; and in others, though the treatment had been hitherto satisfactory, the painful symptoms having been entirely relieved, a sufficient period had not elapsed to enable me to judge of the ultimate result. In two of these cases, the relief afforded by the truss to the distressing symptoms occasionally attendant on the disease, was so immediate and so great, that I am led to give them in detail." P. 263.

We regret that the space we have already devoted to our notice of this volume does not admit of our quoting these two interesting cases. We can only observe that they show, in a forcible manner, the great benefit that may be derived from the application of pressure in painful cases of this disease. Our author remarks—

"Patients afflicted with varicocele in early life, often labour under a degree of mental distress very much out of proportion to the actual disease. These hypochondriacal symptoms are partly owing to the dyspepsia so commonly coexisting with this complaint, and partly to an apprehension, by no means unfounded, of the disease impairing the nutrition of one of those organs which exercise a marked influence on the characters of the sex. By appropriate general treatment and encouraging advice, combined with local treatment, the painful feelings alluded to may generally be removed. In other instances, the uneasiness in the testis and spermatic cord, and even in the loins, is so great as to produce much real suffering, and to prevent the person affected from making any kind of exertion. In Case 4, which was an instance of this kind, the patient was prepared to submit to an operation, had I recommended one, but the benefit derived from the truss was sufficient to render so severe an alternative unnecessary. In this case, the distention of the veins was so slight, that I think the relief obtained may be in some measure due to the pressure made on the spermatic nerves." P. 265.

Mr. Curling believes that but little attention is paid to constitutional treatment in varicocele, "which is commonly regarded as exclusively a local disease. In the class of cases in which the benefit derived from pressure is most apparent, the subjects of the disease are persons between 18 and 30 years of age, of weak frame and constitution, and subject to dyspepsia, and whose venous system and circulation are feeble, as is evinced by the large size of the superficial veins, particularly in the lower extremities, paleness of the countenance, and cold hands and feet. In these cases, the operation of local remedies may be aided materially by general treatment, such as the exhibition of steel and quinine, a nutritious diet, sea-bathing, and similar measures, calculated to improve the tone of the system." Mr. Curling notices the liability of the complaint to return unless the causes producing it are avoided, and for this reason he advises the use of the truss for some time after all symptoms of the affection are removed. He adverts to another class of cases in which the application of pressure is capable of giving considerable relief though not of curing the disease. They are cases occurring at a somewhat advanced period of life, with which most practitioners are familiar. In these cases the application of pressure "not only removes the slight uneasiness which exists when the veins are pendent, but also counteracts the tendency to further dilatation, though the enlargement is too great to admit of the vessels being reduced to their former size." Our author adds—

"From these observations it will appear that I consider the treatment by pressure to be applicable either for the cure or relief of the majority of cases of varicocele occurring in practice. Certainly in all those cases in which tolerably firm pressure, with the fingers, at the abdominal ring, removes the sense of weight and uneasiness along the cord, this plan may be tried with every prospect of a beneficial result; and its simplicity, freedom from all risk, and efficiency, in my opinion, render it superior to every other mode of treatment that has hithero been resorted to." P. 267.

In all the cases of varicocele which Mr. Curling has treated by pressure, he has employed the moc-main lever truss, which seems better adapted to make the necessary pressure on the spermatic veins at the external abdominal ring than any other instrument that he knows of. It is not liable to shift, and, what is very important, the degree of pressure can easily be regulated by the patient. He has used it with success in cases where the patient has tried other trusses without obtaining relief.

"The truss should be applied so as to make rather firm pressure: it often happens that, though worn with comfort after being adjusted in the morning, towards the after part of the day it begins to produce uneasiness. When this is the case, the pressure may be diminished. In general, the truss need be worn only during the day, though in some instances I have thought it advisable to recommend its use during the night also. Thus in one case the patient suffered uneasiness in lying on the side affected, and was able to pass a better night on wearing the truss. When the scrotum is unusually pendulous, or when the veins are very long, and form a plexus of any size, I advise the addition of the silk net suspender, which may be readily adjusted to the truss." P. 268.

There are few practitioners who have not been frequently disappointed with the methods of treatment ordinarily resorted to in cases of varicocele. In early life, support often fails in giving relief and preventing the progress of the disease and wasting of the testicle; and operations on the veins cannot be performed without risk to the patient. If, therefore, pressure on the veins be capable of affording all the benefit represented in this paper, the practice must be regarded as a valuable improvement in surgery. At first sight it appears opposed to the received notions of the indications to be fulfilled in the treatment of varicocele, but the modus operandi has been satisfactorily explained by Mr. Curling, and our preconceived theories must yield to the fruits of experience.

XVII. On a particular Derangement of the Structure of the Spleen. By *J. B. S. Jackson*, M.D., Boston, U.S.A. Communicated (with some Remarks and Comments) by *Thomas Hodgkin*, M.D.

In this paper, Dr. Hodgkin calls attention to a partial, well-defined alteration in the texture of the spleen, described by him in the 17th volume of the Transactions, which he was induced to attribute to the extravasation of blood caused by local injury, but which Rokitanski regards as one of the effects of endocarditis, and as produced by the poisoned condition of the blood. He there gives the substance of some communications from Dr. Jackson of Boston, containing his observations on this derangement. As neither Dr. Hodgkin nor Dr. Jackson seem to have arrived at any satisfactory conclusion on the subject, we shall not dwell further on this paper, and we think that one containing views as yet crude and imperfect might have been omitted without impairing the value of the Transactions.

XVIII. On a Luminous Appearance of the Human Eye, and its Application to the Detection of Disease of the Retina and Posterior Part of the Eye. By *William Cumming*, late House-Surgeon to the London Hospital.

Mr. Cumming, after giving some extracts from the works of Beer and Tyrrel, pointing out the cases in which reflection from the posterior part of the human eye has been observed, states that he has not found or heard of any author who has described a reflection from the posterior part of the perfectly-formed and healthy eye of the human subject. The object of this paper is to shew "that the healthy human eye is equally, or nearly equally, luminous as the eye of the cat, dog, &c., when observed under favourable circumstances, and the application of the abnormal appearance, or want of, this luminosity, to the detection of changes in the retina and posterior part of the eye."

Mr. Cumming states that the reflection may be seen in the following manner :—

"Let the person whose eye is to be examined be placed at the distance of ten or twelve feet from a gas or other bright light; the rays of light must fall directly on his face; all rays passing laterally of his head must be intercepted by a screen, placed half way between the light and the eye examined. If the reflection be bright, it will be at once seen from any spot between the light and the screen.

"The following observations were made in two rooms; in one of which was a gas-light, the other completely darkened. The person whose eye was to be viewed was placed in the dark room, five feet from a half-closed door opening into this room; he directly faced the light, also at the distance of four or five feet from the door.

"The appearance of the reflection was in most cases extremely brilliant when seen from a position between the door and light. In some it was at once obvious with the door wide open; in others it was seen with great difficulty, and not till every ray of light passing to the side of the iris was carefully intercepted by the door on one side and the hand or a book on the other. The reflection was always seen much more readily and brilliantly when the eye was turned slightly to the side, and the rays of light passed through the pupil obliquely. On passing to the other side of the door, the luminosity was seen with greater difficulty. In this position it is necessary to have the eye turned to the side, to exclude all rays by the hand except those passing directly to the eye. In this way the reflection may be seen distinctly at the distance of eight inches.

" In the majority of cases, however, it may be seen as follows: Let the person under examination sit or stand eight or ten feet from a gas-light, looking a little to the side; standing near the gas-light, we have only to approach as near as possible to the direct line between it and the eye to be viewed, at once to see the reflection. Or, in a dark room, a candle being placed four or five feet from the eye, if we approach the direct line between them we shall be able at once to see it in many cases. If solar light be admitted through a nearly-closed shutter into a dark room, the luminosity may be seen when the pupil is tolerably dilated, the patient standing five or six feet from the aperture, and the observer occupying the position before indicated.

" These then are the circumstances necessary for seeing the luminosity. a. That the eye must be at some distance from the source of light; the distance being greater in proportion to the intensity. b. That the rays of light diffused around the patient (and sometimes around the eye itself) should be excluded. c. That the observer should occupy a position as near as possible to the direct line between the source of light and the eye examined; hence it is sometimes necessary for the observer to stand obliquely, that his eye may approach nearer to the direct line.

" The appearance of the reflection itself not only varies much in colour and intensity in different persons, but also from the circumstances under which it is seen, viz. the greater or less intensity of light, the position of the eye examined, and the distance at which it is viewed.

" When the reflection is seen under the influence of a dim light, as that from a candle, or a few solar rays, a red lurid glare, like that from a dull coal fire, is observed, evidently proceeding from the bottom of the eye, and, though not distinctly concave, yet conveying the idea of concavity. The character of the reflection thus seen by a faint light, at the distance of two or three feet, is very uniform, and does not present much variety of tint.

" When the eye receives rays from a good bright light ten feet distant, and we stand near the light, the reflection is then seen extremely brilliant; presenting a fine metallic lustre, and varying from a bright silver or golden to a decided red tint: the latter being the more usual colour. While viewing the reflection at this distance, it sometimes undergoes a distinct change, suddenly altering from a copper or red colour, to a silver tint: this happens sometimes in consequence of a slight movement of the eye, but not unfrequently is observed without any movement having taken place.

" Although the reflection is more readily seen in an eye with a large pupil, its lustre does not depend upon this circumstance. In two eyes with pupils of equal diameter, the intensity of the reflection frequently varied greatly. In one case, in which the reflection was very dusky in appearance, and the pupil small, atropine was dropped into the eye. I then observed that, though the extent of luminosity was increased, it still retained the same dusky hue. The greater facility with which the reflection is seen when the eye is directed slightly away from the light, appears to depend on the more patulous condition of the pupil.

" On approaching within a few inches of the eye, the reflection is not visible, for, before our eye can be brought within range of the reflected rays, the incident rays of light are excluded. On placing before the eye examined, a black card with an aperture the size of the iris, the intensity of the reflection was observed to be somewhat diminished.

" In cases in which the lens had been removed, the reflection was indistinct at a distance, but was rendered somewhat clearer by the aid of a double convex lens placed before the eye examined; but at two or three feet distant, the reflection was as obvious as in cases in which the lens was present.

"Among the cases I have examined, I have recorded indiscriminately the appearance of the luminosity in twenty persons with good and perfect vision, whose ages varied from a few months to sixty years. In sixteen cases the reflection was bright and very evident; in four, faint, and seen with difficulty; and in one it was not seen at all; in the last case, the pupils remained small in the shade. If these observations are confirmed by other observers, we may say that the reflection ought to be seen in every healthy eye with a good-sized pupil." P. 287.

Mr. Cumming next endeavours to shew the source or cause of the reflection, which he ascribes to the choroid with its pigment. But, while he regards this as the principal reflecting structure, he believes that its effect is increased by the light returned from the retina and concavity of the hyaloid body. The reflection from these structures would be considerably increased in brilliancy, from the concentrating influence of the concave shape of the retina, and the focal distance of the lens. Our author makes some interesting observations on cat's-eye-amaurosis, and is inclined to think that two different things have been confused under this name—that, in the majority of cases in which the normal luminosity of the eye was observed, the reflection exactly corresponding with that described in this paper; and that the others, in which red vessels and a margin to the reflecting surface were seen, were cases of deposit of lymph in the retina. He states—

"If this be a correct analysis of these cases, the mystery that hangs about cat's-eye-amaurosis vanishes; the first class of cases were cases of amaurosis arising from cerebral and other causes, in which, the retina and choroid being perfect, the normal reflection from them was seen; the second division consists of cases of deposit of lymph or other substances in or about the retina. It is then at once evident that a mere luminosity of the eye will in no case be a sign of altered condition. It will be necessary first to become acquainted with the normal reflection—its modifications in different lights and positions, and at various periods of life, and in persons of a dark or fair skin; then by the detection of an altered condition of such reflection (and assisted in many cases by contrast with the opposite eye), or by its entire absence, we possess a means of diagnosis in retinal and choroidal disease." P. 293.

In confirmation of its value, as a means of detecting changes in the retina, Mr. Cumming relates four cases. The first was one of imperfect vision of the left eye. The probability was "that it was an affection of the retina; but there was no other sign on which to rely but the statement of the patient with regard to the appearance of scintillations. The pupils were equally black, and the appearance of the eye normal; by this mode of examination it was rendered clear that the left half of the retina had undergone considerable change, and probably its power of transmitting the influence of rays onwards to the cerebrum." The other three cases are equally interesting, as showing the utility of this mode of detecting alteration in the retina. The fourth was one of cerebral amaurosis in which the reflection was perfect.

This is a communication of considerable value and interest, and highly creditable to the author. In establishing the fact of the normal luminosity of the eye, Mr. Cumming has discovered a mode of discriminating diseases of the posterior part of the eye which must prove of great service in many cases of difficulty.

XIX. Account of a Case in which an Abscess in the Neck communicated by an Ulcerated Opening with the Arch of the Aorta, and in which the Hæmorrhage did not prove fatal in less than Forty-eight Hours. By *George Busk,* Surgeon of the Hospital Ship " Dreadnought."

The title of this paper is liable to mislead. The abscess should have been termed *fistulous.* The patient was a woman about thirty-five years of age. An abscess in the neck, originating apparently in an enlarged gland, burst about four months before her death, and had continued to discharge matter ever since. Mr. Busk remarks—

" The case is chiefly interesting as affording an unequivocal instance of a communication being formed between the cavity of an abscess and a large arterial trunk, in consequence of an ulcerative process being set up from without, and going on to produce such a thinning of the arterial tunics, that they finally give way under the impetus of the blood. It is evident that, had this communication been set up at an earlier period, and before the bursting of the abscess, it would have been very difficult, if not impossible, at that stage, to have avoided mistaking the abscess for an aneurism: for when, towards the end, the orifice was closed, and the cavity of the abscess filled with blood, such a pulsation was caused, as very strongly to simulate that presented by an aneurismal tumour." P. 301.

The extension of ulceration from a *fistulous* abscess to a large arterial trunk, as in this case, is not so rare an event as Mr. Busk supposes. The communication, however, of chronic abscess with a large artery by ulceration so as to simulate aneurism is extremely rare, so rare, indeed, that we know of no unequivocal case on record.

XX. On the Relation between the Constituents of the Food and the Systems of Animals. By *R. D. Thomson,* M.D.

The author, in the present paper, gives the results of experiments on the amount of wax and oil contained in the food of two cows, as compared with the quantity of both of these found in the milk and dung. The details have been already printed in other treatises; to which he refers in the one now before us.

As it is evident then, that the present essay is itself an epitome of other works, the business of analysing it becomes more difficult. The first table informs us of the fact that cows taking certain food or foods, grass being one of them, give out much less oil and wax in their butter and dung than has been supplied to them in their food. That, on the contrary, when fed on other substances, hay being always one of them, they give out more oil and wax in their butter and dung than they have received in their food. We have no explanation offered of the cause of this difference, but an inference drawn that the oil in the food alone is not adequate to the production of the oily matter in the cow; an inference which seems deduced from the latter to the exclusion of the former result just mentioned.

In another experiment, the carbon, hydrogen, nitrogen, and oxygen contained in the solid parts of the food and dung of a stall-fed cow were separately ascertained, and the difference estimated as the consumption of the animal. From the quantity of nitrogen thus consumed is deduced the amount of nitrogenous or nutritive matter assimilated by the animal, the rest being considered as forming the calorifiant portion of the food absorbed, and it is found that the former is to the latter as 1·560 to 13·100, or as 1 to $8\frac{1}{3}$ very nearly. Milk, as stated by the author, contains one part of nutritive to two parts of calorifiant constituents. Now, these may probably be taken as the extreme limits of the proportions which should be borne by the quantities of these respective principles when mixed in the diet. The growth of the young animal, and the demands arising from exercise of the muscular system in all requiring an increase, a state of rest admitting a diminished proportion of nutritive material.

Tables follow exhibiting the per centage of albuminous matters in a number of articles of domestic use as they occur in commerce, and also the relation of nutritive to calorifiant matter in many important items of diet. These are interesting and valuable, but we cannot extract, and it would be unfair to curtail, them. Another table gives the result that grass affords the best products when given to milch cows, although the animals receive less nitrogen when fed upon it than in many other cases; and the paper concludes with the remark that, the preceding observations lead to an extensive field of experiment and deductions of a highly practical nature, and may assist in indicating the direction in which the physician should pursue his enquiries when studying the laws by which the animal system is to be retained in a state of health. In all this we concur, considering the whole essay as offering some useful suggestions rather than pointing out any principles which are deserving of full confidence.

We do not much like the tone of this paper: there is the appearance of putting too much on the author's own experiments, and referring too little to any other sources of knowledge. Thus, the author, having tabulated the results of feeding two cows on different articles, writes thus, "we may infer from these results, that grass affords the best products, because the nutritive and calorifiant constituents are combined in this form of food in the most advantageous relations." Now, if the circumstance to be explained were a well-established fact, some value might attach to the proffered explanation; but, as compared with the vast accumulated experience of dairymen and others, how inadequate is this petty experiment to show us what kind of food causes cows to yield most milk and butter. As the case stands, we have merely a conjectural explanation of an inference founded on insufficient observation. We say conjectural explanation, for the Table only shows that the products in milk and butter do not increase with the quantity of nitrogen in the food.

At page 337 we have the following extraordinary passages.

"From this Table, likewise, we infer that, as nature has provided milk for the support of the infant mammalia, the constitution of their food should always be formed on the same model. Hence we learn that milk, in some form or other, is the true food of children, and that the use of arrow-root, or any of the members of the starchy class, where the relation of the nutritive to calorifiant matter is as 1 to 26 instead of being as 1 to 2, by an animal placed in the circumstances of a human infant, is opposed to the principles unfolded by the preceding Table."

Now, how can a Table, which shows only the proportions between nutritive and calorifiant matters in milk, and several other alimentary substances, furnish the inference that the constitution of infant food should always be formed on the same model? Reflection on what we observe in Nature may teach us this, but Dr. Thomson's Table never can, albeit a good Table and teaching us an important condition existing in this model. We opine, too, that the discovery that milk is the true food of children has a date somewhat anterior to that of the construction of the Table in question. The Table unfolds no principles, but shows that the starches differ in an important particular of their constitution from the matters contained in milk. It is certain, therefore, that the starches alone cannot form an analogous diet; it is probable that the use of them as substitutes is not advantageous. We give the author all credit for the skill and labour displayed in his chemical researches, but wish to remind him, in conclusion, that, for the promotion of knowledge, legitimate inferences are as much needed as trustworthy experiments.

We here close our notice of this volume of the Transactions, which, with a few exceptions, fully sustains the high reputation of the Society. Often as we have had occasion to express our approbation of these annual volumes, we have seldom performed the task with greater pleasure than in the present instance.

